# Critical care nurses’ experiences of caring challenges during post-resuscitation period: a qualitative content analysis

**DOI:** 10.1186/s12912-024-01814-2

**Published:** 2024-03-03

**Authors:** Mahnaz Zali, Azad Rahmani, Hadi Hassankhani, Hossein Namdar-Areshtanab, Neda Gilani, Arman Azadi, Mansour Ghafourifard

**Affiliations:** 1 Nursing faculty, Tabriz University of Medical Sciences, Tabriz, Iran; 2 Health faculty, Tabriz University of Medical Sciences, Tabriz, Iran; 3 Nursing faculty, Ilam University of Medical Sciences, Ilam, Iran

**Keywords:** Cardiopulmonary resuscitation, Nursing care, Qualitative research, Post-resuscitation

## Abstract

**Background:**

Patients in the post-resuscitation period experience critical conditions and require high-quality care. Identifying the challenges that critical care nurses encounter when caring for resuscitated patients is essential for improving the quality of their care.

**Aim:**

This study aimed to identify the challenges encountered by critical care nurses in providing care during the post-resuscitation period.

**Methods:**

A qualitative study was conducted using semi-structured interviews. Sixteen nurses working in the intensive care units of three teaching hospitals were selected through purposive sampling. The Data collected were analyzed using qualitative content analysis.

**Results:**

Participants experienced individual, interpersonal, and organizational challenges when providing post-resuscitation care. The most significant challenges include inadequate clinical knowledge and experience, poor management and communication skills, lack of support from nurse managers, role ambiguity, risk of violence, and inappropriate attitudes of physicians towards nurses’ roles. Additionally, nurses expressed a negative attitude towards resuscitated patients.

**Conclusion:**

Critical care nurses face several challenges in providing care for resuscitated patients. To enhance the quality of post-resuscitation care, address the challenges effectively and improve long-time survival it is crucial to implement interventions such as In-service education, post-resuscitation briefing, promotion of interprofessional collaboration among healthcare teams, providing sufficient human resources, clarifying nurses’ roles in the post-resuscitation period and increasing support from nursing managers.

## Introduction

Post-resuscitation care, which starts as soon as spontaneous circulation returns, is an important factor in the long-term survival of cardiac arrest victims [1, 2]. High-quality post-resuscitation care includes special care based on the complex pathophysiological processes that occur before, during, and after cardiac arrest [3]. Providing such care requires teamwork, coordination, and collaboration among all healthcare providers involved in post-resuscitation care [4]. Nurses play a vital role during this time [5] as they not only assess and manage patients’ responses to health problems but also verify and implement some interventions/medications prescribed by other healthcare professionals [6]. 

Resuscitated patients require specialized care and are often admitted to intensive care units [7]. Critical care nurses encounter important decisions, stressful situations, and numerous ethical dilemmas, [8] while facing significant physical and psychological pressure [9]. Consequently, there are several challenges associated with providing high-quality care [10, 11].

Some common challenges include insufficient consideration nurses’ opinions by physicians, [12] poor teamwork and inter-professional cooperation, [13] inadequate involvement in care planning, [14] subpar mental and physical well-being, [15] lack of support from the organization, [16] time pressure, limited availability of necessary resources/facilities [17] and ineffective in-service training [18]. These challenges have led to high levels of occupational stress [19] and burnout, [20] among critical care nurses resulting in many of them considering leaving intensive care units [21]. 

There is a lack of relevant studies investigating the challenges encountered by critical care nurses in providing post-resuscitation care. Given the critical condition of resuscitated patients that requires high-quality nursing care, it is crucial to investigate the challenges that critical care nurses face in caring for these patients.

### Aim

This study aimed to identify the challenges encountered by critical care nurses in providing care during the post-resuscitation period.

## Methods

### Design and setting

This qualitative study was conducted at three teaching hospitals affiliated with Tabriz University of Medical Sciences from November 2021 to February 2022. These hospitals collectively have a total of 26 intensive care units and serve as a referral centers for patients with critical conditions in the East Azerbaijan Province, located in northwest Iran. The guiding research question was as follows: What challenges do critical care nurses encounter while providing care for resuscitated patients?

### Participants and sampling

Sixteen nurses, 11 (68.75%) females and 5 (31.25%) males, were selected using a purposeful sampling method. Participants were from general, burn, surgical, poisoning, and medical intensive care units. The inclusion criteria were as follows: at least two years of clinical work experience in intensive care units, extensive experience in post-resuscitation care, and after expressing the willingness to participate in this study. In each educational center, a research assistant identified potential participants and informed them about the study and its purpose. Once the participants expressed their willingness to participate in the study, this information was communicated to the main researcher (M.Z). Subsequently, the she (M.Z) contacted these participants and provided a comprehensive explanation of the study’s purpose and methodology. Finally, she obtained written consent from them. The mean age and work experience of the participants were 39 ± 37/6 years and 12 ± 6/42 years, respectively. The first two participants had more than ten years of experience working in intensive care units. Others were selected based on an analysis of previous interviews to fully understand the participants’ perspectives.

Sampling was purposive, trying to select the maximum diversity in terms of nurses’ age, gender, and education level. Sampling continued until data saturation reached the point where new concepts did not emerge or where there was no significant development of previous concepts.

### Data collection

Data were collected through semi-structured, face-to-face interviews conducted by a principal researcher (M.Z, Female), experienced in qualitative research. All invited nurses participated. Interviews were conducted in agreed locations- fourteen in private setting within the selected hospitals and two at main researcher’s workplace. The private areas within the hospitals included nurses’ break room or the rooms provided by head nurses. The aim was to create a private, comfortable setting for the participants to share their experiences. To ease stress, we first discussed their education and professional background. The main research questions were as follows: What kind of care do you provide to your patients in the post-resuscitation period? What problems do you face when providing such care? Why have these problems arisen? What are the barriers to and facilitators of providing post-resuscitation care in the workplace? Who supports you in providing such care? Further probing questions were asked to clarify ambiguities according to the participants’ responses to these questions. For example; Who? How? Where? Would you explain this in more detail? Please provide some examples of this point. Additional interviews were conducted with five participants, selected by M.Z and A.R after the initial analysis to clarify ambiguities, provide further explanation and fully comprehend key codes. These five participants were informed of their right to refuse the follow-up interview. All of them agreed to proceed. Three interviews took place in hospitals, and two were conducted at the main researcher workplace. These interviews were conducted after the initial interview with the each participant. In fact, the next participant was not interviewed until the data from one participant was fully collected and analyzed. The Interviews were conducted in Persian or Azeri based on the participants’ convenience. On average each interview lasted 45 min.

### Data analysis

The collected data were analyzed using Graneheim and Lundman’s method (2004) for qualitative content analysis [22]. The interviews were analyzed immediately after each interview. First, the interviews were transcribed verbatim and then read and re-read several times to achieve an overall understanding of their contents. The meaning units were words, sentences, or paragraphs from the interviews. The meaning units were abstracted and labeled with codes (387 codes). The initial codes were condensed, interpreted, and compared with other codes by considering their similarities and differences, and the categories and subcategories were identified categories and sub-categories were then identified through this iterative process. Agreement on the final categories and sub-categories was reached through three meeting involving four researches.

The rigor of the study was established according to Lincoln and Guba’s (1985) criteria for: credibility, transferability, dependability, and confirmability [23]. Credibility was ensured through prolonged engagement of the main researcher (M.Z.) in the research setting and joint meetings with co-researchers to discuss findings. We also utilized the member checking method to verify the accuracy of extracted data and codes or make necessary modifications. After coding each interview, the coded interviews were shared with participants to ensure the correctness of their codes and interpretations. Corrections were made when there was a mismatch between researches interpretation and participants’ point of view. Transferability was ensured by selecting samples with maximum variation in terms of personal and occupational characteristics. Dependability was achieved through the involvement of four researchers in the data analysis process. Confirmability was enhanced by documenting the details of the data collection, analysis, and interpretation.

#### Ethical considerations

This study was approved by the Regional Ethics Committee of the Tabriz University of Medical Sciences (Code No: IR.TBZMED.REC.1399.1035) and was conducted in accordance with the Helsinki declaration. Written informed consent was obtained from all participants who were also informed about the study plan, their right to not participate and the confidentiality of their information in both oral and written forms. All research analyses and reports were conducted while maintaining the confidentiality of participants’ identifying information. In this report, participants are referred to by numbers without including any identifying information. Regarding follow- up interviews, no additional approval was required to conduct them as per guidelines provided by the Regional Ethics Committee.

## Results

Three major themes, namely individual, interpersonal and organizational challenges faced by nurses in providing post-resuscitation care emerged from the analysis (Table 1).

**Table 1:** Description of themes and sub-themes resulting from the study

Themes	Sub-themes
Individual challenges	Lack of knowledge
	Non-reflective experience
	Negative attitudes
	Poor managerial skills
Interpersonal challenges	Communication avoidance
	Physician dominance
	Fear of violence
Organizational challenges	Lack of managerial support
	Role ambiguity

### Individual challenges

Some of the challenges faced by critical care nurses in providing post-resuscitation care are attributed to individual experiences. This theme comprises four sub-themes: lack of knowledge, non-reflective experiences, negative attitudes, and poor managerial skills.

#### Lack of knowledge

Lack of knowledge emerged as a significant challenge faced by nurses during the post-resuscitation period. Participants highlighted their insufficient understanding of patient pathology, complications arising from resuscitation, management and assessment of vital systems, and utilization of medications. This knowledge gap among critical care nurses can be attributed to various factors, such as inadequate coverage of relevant topics in university curricula, limited access to continuous education courses and suboptimal implementation of up-to-date guidelines within hospitals. Additionally, Participants expressed difficulties in accurately assessing patients’ conditions, and acknowledged that much of their care relied on routine practices rather than incorporating the latest research findings.

“Knowledge is the foundation. When you lack knowledge, you don’t know what is happening or what will happen in the future. You may not provide the necessary care” (P.2).

“I had some colleagues who struggled to understand patients’ current condition or accurately identify changes due to a lack of knowledge. As a result, they failed to recognize problems in a timely manner. We have not received proper training or guidance on how to provide specialized post- resuscitation care” (P.11).

#### Non-reflective experience

Limited and non-reflective experiences pose additional challenges for nurses in post-resuscitation care. Non-reflective experiences refer to repetitive encounters without reflection or correction. Participants highlighted that despite their extensive years of working in Intensive Care Units (ICUs) and caring for numerous post-resuscitation patients, they did not find these experiences useful due to the lack of reflection. They believed that frequently repeating non-evidence-based and unprincipled care hindered their ability to provide high-quality care and reduce the chances of survival during the post-resuscitation period. The absence of reviewing previous cases and experiences, particularly through group discussions using effective educational materials, emerged as a significant barrier preventing nurses from engaging in reflective practices.

“Participation in resuscitation and solely providing routine care is insufficient. Nurses need to actively review clinical cases, reflect on why certain events occurred, and identify any essential care that may have been missed. Otherwise, what you label as “care” becomes nothing more than a repetitive act” (P. 3).

#### Negative attitudes

The participants shared that there was a prevailing negative attitude among nurses towards resuscitated patients. Many critical care nurses believed that these patients had little chance of survival and would eventually pass away. Furthermore, they viewed these patients as educational cases for nursing or medical students. This negative mindset discourages nurses from providing optimal post-resuscitation care. Consequently, there has been a significant decline in the quality of nursing care provided. According to the participants, these negative attitudes pose a major challenge in providing post-resuscitation care. Nurses also emphasized that such attitudes have become ingrained within the culture in intensive care units, where having pessimistic thoughts is not uncommon.

“Nurses generally think that a resuscitated patient would no longer survive. They feeling hopeless and often waiting for the patient’s next cardiac arrest” (P.12).

#### Poor managerial skills

Another challenge highlighted by the participants was the poor managerial skills of nurses. Ineffective time management and failure to prioritize patients’ problems can result in crucial issues being overlooked and valuable time for providing high-quality care being lost. Additionally, nurses faced difficulties in accessing timely support from their colleagues or nursing managers, such as clinical supervisors, when needed. Often, they encountered obstacles in seeking assistance during critical moments or were unable to receive prompt help. Furthermore, participants reported challenges in obtaining timely and adequate equipment necessary for patient care.

“Many nurses spend a lot of time with patients, but they do not always prioritize their essential needs. As Nurses, we should be able to manage our time better. Sometimes we waste time on unimportant things and end up missing out on providing necessary cares” (P.9).

### Interpersonal challenges

Three sub-themes emerged within this theme: communication avoidance, physician dominance, and fear of violence.

#### Communication avoidance

Participants reported instances where nurses attempted to avoid communicating with physicians regarding resuscitated patients. As a result, crucial information about the patient’s situation may not have been relayed to the physician, leading to delays in follow up care. According to participants, these reluctance wasn’t just limited to communicating with physicians. Nurses also exhibited sparse communication with their colloquies unless absolutely necessary. There is a lack of desire for provide information about resuscitated patients. This reluctance may stem from several factors. First, some nurses view these patients as unlikely survivors, leading them to label such cases as “dying” or “resuscitated”. This perception fosters sense of futility and devaluation of the resuscitated patients. Second, Nurses believe that even doctors, supervisors and colleagues share this view which results in less communication about the patient’s condition. Participants mentioned that doctors might perceive their frequent updates about resuscitated patients as an unnecessary burden. Furthermore, nurses’ limited communication skills and fear of being misinterpreted add to these barriers.

“Some physicians view these patients as “dying” and show no interest in updates. I have experienced this issue firsthand. When you call them about resuscitated patients they react angrily as if you are disturbing them“(P.2).

“I had a colleague who struggled to report to the physician. The patient was unstable, but due to her inability to deliver a clear report, the physician failed to grasp the urgency of the situation and neglected immediate treatment needs.” (P.6).

#### Physician dominance

Participants reported that the dominance of physicians and their lack of attention towards nursing care were other challenges in providing post-resuscitation care. Nurses consistently pointed out that due to the inappropriate and unexpected behaviors of physicians, they have lost their motivation to actively participate and take a leading in post-resuscitation care. They also mentioned that some physicians adopt a top-down approach, treating nurses as inferior. As a result, they disregard nurses’ input and exhibit inappropriate behavior. Some participants even stated that physicians attempt to interfere with independent nursing care and restrict the authority of nurses.

“Nurses often have valuable insights and opinions about patient care, but some physicians dismiss their views because they look down on the nurse. There are doctors who do not consider nurses as highly skilled and educated professional resulting in the disregard of their opinions.“(P.9).

#### Fear of violence

Nurses are prone to workplace violence because of the high responsibilities and additional stress imposed by the patients’ family members. Furthermore, during the post-resuscitation period, nurses may also face instances of violence from other medical staff members. Nurses often experience workplace violence in the form of aggressive explicit criticism and insults. This can occur horizontally from nursing colleagues or vertically from physicians or nursing managers and most violence is verbal in nature. However, there have been a few cases where nurses have also encountered physical violence from family members.

This situation arises due to the lack of understanding about the patient’s condition and the heightened stress experienced by family members. The resulting violence can instill fear in nurses, leading them to be hesitant in involving family members or providing information about a patient’s condition. Some participants stated that incidents of violence are more prevalent during the post-resuscitation period compared to other critical conditions, attributed to factors such as high acuity of the patients, intense concern from family members, and increased workload for the healthcare personnel.

“In some cases, nurses may even insult each other, questioning why certain care tasks are being performed. This creates additional pressure as supervisors have similar expectations from all staff members. It is indeed a challenging situation for everyone involved.“(P.16).

“During the resuscitation, family members are often not present. However, when they arrive and witness the condition of their loved one, their emotional response can increase the likelihood of violent behavior. Managing such situations becomes extremely challenging “. (P.5)

### Organizational challenges

Two sub-themes were identified within this theme: lack of managerial support and role ambiguity.

#### Lack of managerial support

Lack of support from nurse managers has been identified as one of the significant organizational challenges during the post-resuscitation period. Nursing managers expect nurses to strictly follow physician orders without providing input on post-resuscitation care plans, resulted in limiting nursing authority and increasing physician interventions in nursing care. Additionally, nurse managers make minimal effort to reduce workload and expect nurses to handle all resuscitated patients as well as other cases without assistance. Interestingly, if nurses are unable to manage this heavy workload, they are accused of being incapable of providing adequate nursing care.

“Resuscitated patients often rely heavily on nurses for care. Unfortunately, managers have not adequately addressed this issue and expect nurses to handle it without assistance. As a result, the quality of care may be compromised and even be neglected at times.” (P.13).

#### Role ambiguity

Participants reported experiencing role ambiguity in post-resuscitation care, as they were unsure about their specific duties, which actions they could in dependently perform, and when they needed physician’s order. This uncertainty leaves nurses hesitant to take any action without physician’s prescription during the post-resuscitation period due to fear of potential legal repercussions. This becomes even more critical when the attending physician delays providing necessary orders, resulting in a lack of care for the patient during this time. This issue is particularly pronounced in teaching hospitals where certain tasks are delegated to medical students.

“I’m unsure about what tasks are within my scope of practice and what are not. The duties or responsibilities have not been clearly defined which is where the problem and confusion originate. For example, while reliable sources indicate that certain tasks fall under the nurses’ responsibilities, some physicians oppose or resist this notion. As a result, many nurses choose to refrain from taking action instead of engaging in arguments.” (P.12).

## Discussion

This study aimed to identify the challenges faced by critical care nurses when providing care during the post-resuscitation period. To our knowledge, this is the first study to address these specific challenges in post-resuscitation care provided by critical care nurses. Therefore, we primarily compared our results with those of studies focusing on resuscitation and ICU care.

Our results suggest that a lack of adequate clinical knowledge and experience poses a significant challenge for nurses in providing post-resuscitation care. The European Resuscitation Council and the European Society of Intensive Care Medicine have published guidelines in post-resuscitation care in 2021 (latest edition) [24]. However, some studies showed that previous versions of these guidelines have not been fully implemented or followed in clinical settings [25]. The importance of clinical knowledge and skills as essential competencies for nurses has been well-documented in previous studies [26, 27]. Nurses are often the first healthcare professionals to arrive at the patient’s bedside after a cardiac arrest and play crucial role in providing post-resuscitation care [28]. Therefore, it is imperative for nurses to have sufficient knowledge about cardiopulmonary resuscitation and post-resuscitation care. The Edelson et al. (2014) study conducted in the United States found that inadequate training of healthcare providers; nurses in particular, pose a significant challenge in improving the quality of CPR (cardiopulmonary resuscitation) [29]. Additionally, increasing nurses’ knowledge and education can lead to better outcomes for resuscitated patients [30, 31].

A lack of reflective experience is another challenge during the post-resuscitation period. The nurses who participated in this study had extensive experience caring for resuscitated patients but did not find it beneficial. They believed that these experiences were not reviewed, and lacked reflection. Reflection serves as fundamental strategy for nurses’ professional development and enables them to learn through clinical care [32]. Results from a previous study showed that monthly case reviews is a key strategy for increasing nurses’ ability to successfully resuscitate [33].

Furthermore, the negative attitude of nurses and the healthcare system towards resuscitated patients was one of the major challenges in providing post-resuscitation care. Some nurses consider these patients to be educational cases that will ultimately die, and managers do not give them priority. Previous studies have demonstrated that healthcare providers stop caring in the post-resuscitation period in the absence of clear clinical evidence, [34, 35] mostly due to fear of severe and long-term unfavorable neurological outcomes in patients [36]. Interestingly, in one study it was found that 19% of patients who experienced early withdrawal of life support therapies in the post-resuscitation period actually had a chance of survival [37]. in another study, it was shown that nurses’ attitudes towards resuscitated patients significantly affected clinical outcomes [38]. The negative attitudes of nurses and the healthcare system seem to be an important barrier to providing high-quality post-resuscitation care. Notably, no research evidence has been found regarding such negative attitudes towards resuscitated patients among healthcare providers or managers internationally.

In this study, lack of support from nursing managers was another major challenge for nurses in providing post-resuscitation care. Previous research has shown that there is variation between organizations in terms of resuscitation implications [39, 40] several studies have reported that the nurse-patient ratio is an important factor in post-resuscitation care quality [41] and plays significant role in the outcomes of resuscitated patients [42]. Therefore, providing adequately trained staff and transferring resuscitated patients to the ICU are critical for improving patient outcomes, [43, 44] which largely depends on managers’ performance.

Our results also showed that nurses were less willing to communicate with other nurses, physicians, and patients’ family members during the post-resuscitation period. This is mainly due to poor communication skills, inappropriate physician behavior, and fear of violence. This reluctance to communicate leads to a significant decline in the quality of post-resuscitation care as it results in delays or a lack of information about the patient’s condition. Cardiopulmonary resuscitation is a complex process that requires teamwork, inter-professional cooperation, and communication [45]. However, one study showed that even when resuscitation team members work together, they often lack the cohesion of a true team [46]. Previous studies have reported that effective communication is essential for successful resuscitation [47]. Moreover, effective nurse-physician communication is a crucial factor in reducing mortality in ICUs [48]. In an extensive literature review, no study was found that specifically investigated communication skills during the post resuscitation period. Regarding the fear of violence, previous studies have reported that nurses [49, 50] and even staff working in a pre-hospital setting [51] were exposed to violence during CPR, and patients’ family members being identified as the main sources of such incidents. In one study, fear of violence emerged as a significant barrier to family member presence during resuscitation [52]. 

Weaknesses in time management and prioritization of care, along with role ambiguity, are additional challenges were identified by nurses in providing post-resuscitation care. Management skills are considered essential for members of the resuscitation team [47] as they play a key role in team’s success [53] and improving patient outcomes [54]. The importance of prioritization [55, 56] and time management [27, 57] has been emphasized in previous studies. In particular, care prioritization is crucial in post-resuscitation period when patients have multiple problems and the burden of care is high. Regarding role ambiguity, some studies have shown that nurses experience confusion about their role in critical situations such as resuscitation [58] and during the Covid-19 pandemic [59]. In this study, high levels of role ambiguity were observed, leading to passivity and reduced involvement of nurses in resuscitated patient care.

### Limitations

The main limitation of this study is that it only focused on nurses’ experiences, and the opinions of nursing managers and physicians were not considered. To provide comprehensive understanding of the phenomenon, further research is needed to examine the experiences of these groups.Additionally, this study has a methodological limitation that should be taken into account when interpreting its results. The data collection method employed in this study was face-to-face interviews with the participants, which may have caused some participants’ experiences to be under-represent. Incorporating observation alongside interviews can enhance the validity of research findings.

### Implications

The findings of this study demonstrate that improving the quality of nursing care during the post-resuscitation period requires addressing the challenges faced by nurses. Updated post- resuscitation care guidelines, regular clinical case reviews, and training in communication and management skills help enhance nurses’ knowledge in post-resuscitation care and improve their clinical, communication and management skills. Such training may indirectly influence nurses’ attitudes towards post-resuscitation care. The Implementation of an inter-professional training course not only improves the quality of nurses’ teamwork and leadership skills but also influences physicians’ attitudes. Nursing managers should be more supportive of their staff, provide nurses with more autonomy, define their role in the post-resuscitation period, and ensure adequate human resources are available to have a significant impact on the quality of post-resuscitation care.

## Conclusion

Nurses experience many challenges at different individual, interpersonal, and organizational levels during the post-resuscitation period. They lack appropriate knowledge and expertise, sufficient communication, and management skills. Fear of violence, inappropriate behavior of physicians, and uncooperative co-workers resulted in nurses being reluctant to communicate. Lack of nursing management support and role ambiguity is another challenge for nurses. Finally, the negative attitudes of nurses and the health-care system towards resuscitated patients can lead to neglect of patient care. Addressing of these issues is crucial for improving the quality of post-resuscitation care. Moreover, conducting effective training courses to enhance nurses’ knowledge and clinical, communication, and managerial skills, emphasizing the support of managers for nurses, providing sufficient human resources, and clarifying their roles are of high importance in this regard.

## Data Availability

The data that support the findings of this study are available from Azad Rahmani (corresponding author) by reasonable request. Due to the fact that the source of data in this study is a qualitative and confidential interview, it is not possible to send the files of the interviews, but the analysis can be shared without disclosing the identities of the participants.
